# On the Existence in the Texture of Animals of a Fluorescent Substance Closely Resembling Quinine

**Published:** 1866-09

**Authors:** Henry Bence Jones


### On the existence in the Texture of Animals of a Fluorescent Substance closely resembling Quinine.

BY DR. HENRY BENCE JONES, A. M., F. R. S.

[From the British and Foreign Medico-Chirurgical Review, July, 1866.]

The discoveries of science are truly marvelous. Who, a few years ago, would have been bold enough to imagine that fluorescence, the property of emitting light-like fluor-spar when heated, could belong to animal tissues and their fluids under the influence of electrical light? or who could have conjectured that this property is owing to the presence of a substance similar to quinine, so similar, indeed, as to be strictly entitled to the name given it by its discoverers, of quinoidine? Yet this marvel is shown to be a fact in the most convincing manner by Dr. Bence Jones in the paper before us, which was read at the weekly evening meeting held at the Royal Institute (that prolific mother of great discoveries,) on the 23d of March last, and was at the same time amply illustrated by experiments.

Not merely was proof afforded of the similarity of the two by optical phenomena of fluorescence in the spectrum produced by electric light, but also by a close similarity of chemical properties.

The inquiry leading to the discovery of quinoidine, originated in experiments on the diffusion of quinine through the animal tissues, being a continuation of that relative to chemical circulation by œmotic action which we noticed in our Review for Oct. 1865. From the results of numerous and very carefully conducted experments, he comes to the conclusion that quinine when used, that is, taken internally, "goes everywhere," even into the crystaline lens, and that everywhere it goes it meets with the natural fluorescent substance like quinine, which most probably is constantly forming and undergoing oxidation. And, referring to its medicinal effects, he adds:

"The incoming quinine causes a temporary excess of quinine in the textures. Probably it causes a stoppage of the fresh formation of quinine from albumen, a temporary arrest of the changes going on, a transfer of action probably to the quinine introduced, so that with large doses deafness and great prostration and almost imperceptible pulse are produced in man, while in guinea-pigs death even is caused by the extreme prostration. In small doses quinine, probably like alcohol, gives an immediate stimulus when the first chemical action takes place; but soon the quinine retards the chemical changes in the nitrogenous substances, just as alcohol, by its secondary action, retards the chemical changes in the hydrocarbons in the different textures."

From the ascertained facts, to-wit: the results arrived at, the author looks forward to two hopeful prospects—one the explanation and cure of ague, the other the treatment of diseases in parts of the body external to the blood-vessels, such as the crystaline lens. We quote his words in explanation:

"1. Assume that a substance like quinine exists in health in the textures, can its rapid destruction and removal through the action of the marsh-miasm give rise to ague? Does quinine cure ague by furnishing a substance which retards the changes which go on in the textures? And in the well known property of arsenic to preserve organic substances, have we also the explanation of its power in curing ague?

"2. If chemical circulation can carry alkaloids even into the non-vascular tissues, is it not reasonable to suppose that medicines pass through the blood and act on the textures?

"And is it not most probable that they take part in every chemical change that occurs outside the blood vessels, as well as in the blood itself? Still further, may we not expect that among the multitude of new substances which synthetical chemistry is now constantly forming, some medicines may be discovered which may not only have power to control the excessive chemical changes of the textures in fevers and inflammations, but may be able to remove the products of insufficient chemical action, even in those diseases which affect the non-vascular textures, as, for example, in cataract and in gout?"*

